# A New System for Surveillance and Digital Contact Tracing for COVID-19: Spatiotemporal Reporting Over Network and GPS

**DOI:** 10.2196/19457

**Published:** 2020-06-10

**Authors:** Shaoxiong Wang, Shuizi Ding, Li Xiong

**Affiliations:** 1 Department of Emergency Medicine Second Xiangya Hospital Central South University Changsha China; 2 Department of General Surgery Second Xiangya Hospital Central South University Changsha China; 3 Department of Respiratory and Critical Care Medicine Second Xiangya Hospital Central South University Changsha China; 4 Xiangya School of Nursing Central South University Changsha China

**Keywords:** COVID-19, China, mobile health, mobile phones, smartphones, contact tracing, social media, spatiotemporal data, GPS, disease tracking, public health, infectious disease, virus

## Abstract

The current pandemic of the coronavirus disease (COVID-19) has highlighted the importance of rapid control of the transmission of infectious diseases. This is particularly important for COVID-19, where many individuals are asymptomatic or have only mild symptoms but can still spread the disease. Current systems for controlling transmission rely on patients to report their symptoms to medical professionals and be able to recall and trace all their contacts from the previous few days. This is unrealistic in the modern world. However, existing smartphone-based GPS and social media technology may provide a suitable alternative. We, therefore, developed a mini-program within the app WeChat. This analyzes data from all users and traces close contacts of all patients. This permits early tracing and quarantine of potential sources of infection. Data from the mini-program can also be merged with other data to predict epidemic trends, calculate individual and population risks, and provide recommendations for individual and population protection action. It may also improve our understanding of how the disease spreads. However, there are a number of unresolved questions about the use of smartphone data for health surveillance, including how to protect individual privacy and provide safeguards against data breaches.

The recent outbreak in China of the coronavirus disease (COVID-19), an infectious disease caused by a new coronavirus (novel coronavirus [2019-nCoV] or severe acute respiratory syndrome coronavirus 2 [SARS-CoV-2]), has developed into a global public health crisis. The estimated basic reproductive number (R_0_) of 2019-nCoV, or the number of additional cases infected by each case, is 2.2 [1]. This is comparable with severe acute respiratory syndrome (R_0_ 2.2-3.6) [2] and Middle East respiratory syndrome (R_0_ 2.0-2.8) [3], showing the high infectivity of SARS-CoV-2. Early tracing and quarantining of close contacts are critical in cutting off the transmission chain and limiting the scale of any epidemic [4]. The current patient-focused biomedical data-based strategy for epidemic prevention and control focuses on identifying patients by clinical examinations. This assumes that patients will go to a clinical institution for diagnosis as their symptoms worsen and doctors will then trace their contacts, provided that their memories are good enough. However, in a modern society characterized by high-speed mobility and high-dimensional complexity, this patient-focused biomedical data-based strategy does not permit sufficiently prompt tracing and quarantining of potential sources of infection in the population. It can, therefore, fail to halt the spread of the disease [5]. Up to 84% of patients with COVID-19 are asymptomatic or have only mild symptoms [6], and their diagnosis tends to be delayed. It is therefore difficult, if not impossible, to trace and quarantine all patients with latent disease before they infect others. On March 11, 2020, the World Health Organization officially declared COVID-19 a pandemic [7]. Many countries have limited travel and shut down cities, but unpredictable latent infections could worsen disease transmission and overwhelm local medical and social systems. It is much more cost-effective to cut off the transmission chain and protect the majority of susceptible people from infection than to treat numerous patients in hospitals. Developing an effective strategy for precise individual monitoring that can trace close contacts promptly and protect the majority of people without limiting basic city functions is, therefore, becoming increasingly urgent worldwide.

Smartphones are widely used electronic products that can detect characteristics of users, such as spatiotemporal trajectory and social contacts. They are already used in health care through websites and apps such as WeChat, Twitter, and Facebook, opening up a new field in medical and scientific research known as mobile health. Current practice in mobile health mainly involves medical surveys, chronic disease interventions, and health education around infectious diseases [8]. To harness this technology for infectious disease prevention and control, systems must be developed to exploit the core information needed: personal spatiotemporal trajectory data, which can be gathered using a GPS and geospatial artificial intelligence (GeoAI) technologies. A GPS is a satellite-based radio navigation system that can provide real time geolocation and time information to a GPS receiver anywhere on Earth. GeoAI is the combination of artificial intelligence (AI) and geographic information systems (GIS), and has been used in public health [9]. For example, Google Flu Trends was used to forecast state-level trends of seasonal flu epidemics in the United States [10]. We suggest that improving the resolution of monitoring and forecasting could enable accurate contact tracing and precise individual-level protection, both of which are critical in the management of COVID-19. They are also realizable using existing technologies.

We therefore proposed the spatiotemporal reporting over network and GPS (STRONG) strategy, a system integrating GPS and social media via a smartphone app and GeoAI. STRONG is characterized by dynamic involvement of the whole population, including both diagnosed patients in and out of hospitals, as well as apparently healthy individuals. This is important in control of severe communicable diseases with a high R_0_ such as COVID-19. Updated spatiotemporal trajectory data from GPS–enabled smartphones can be collected through routinely used social media apps. The back end system analyzes spatiotemporal data from all users and promptly and accurately traces the close contacts of all patients. Unlike current memory-dependent contact tracing methods, spatiotemporal trajectory data in the cloud can provide unique, detailed, permanent, and traceable spatiotemporal trajectory data for both diagnosed patients and healthy individuals. This permits early tracing and quarantine for potential sources of infection. The massive volume of spatiotemporal data collected can also be merged with other data sources including clinical data from medical institutions and macroscopic data such as GIS data from government sources and, if possible, volunteer-interacting data from the smartphone apps ecological system, like location data deriving from Uber history or payment history. These integrated medical, biological, and demographic data can then be analyzed using AI–based methods including GeoAI to predict epidemic trends on a macrolevel [11]. The system will generate an algorithm to calculate time-specific individual and location risks, and guide personal protective measures. It will also provide important reference information to help governments formulate policies for a protective network for the healthy population. Crucial epidemiological data points, such as the infection’s transmissibility through each route and the role played in transmission by subclinical, asymptomatic, and mild infections, could also be calculated, providing a clearer understanding of the spread of the epidemic than has been possible to date [12].

To put STRONG into practice for COVID-19 prevention and control, our team has developed the WeChat mini-program Geo WeChat AI System (Geo-WAS). WeChat is the most popular multifunctional social media app in China, with 1.15 billion monthly active users, or 80% of the Chinese population, and created about 29.63 million job opportunities in 2019 [13]. It is, therefore, the obvious platform for collection of personal spatiotemporal trajectory and social behavior data, especially in the current emergent epidemic situation in China. A key component of the WeChat platform is the technology of mini-programs, or powerful applets within the WeChat ecosystem [14]. Our mini-program collects data from users’ voluntary WeChat activities, including time and location labels, volunteered smartphone assisted real world activities history over the previous 14 days, and the current maximum incubation period for COVID-19, to generate an updated space-time Quick Response (QR) code to use for identification.

We have also created an individual dynamic spatiotemporal risk index to quantify the real time accumulative exposure risk for each user. The risk index is a ratio of the weighted proportion of locations containing infectious, suspected, and pending users defined as the close contacts of individuals who are either infectious or suspected to be in all areas where the user has been within the last 14 days. The spatiotemporal risk index is calculated using the formula: 
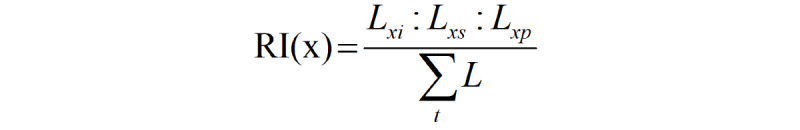
 RI(*x*) is the individual dynamic spatiotemporal risk index for an individual *x* in Geo-WAS; *L_xi_*, *L_xs_*, and *L_xp_* are the number of locations that user *x* has been to in the past 14 days that contain any marked infectious, suspected, and pending users; and 
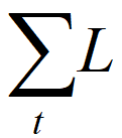
 is the number of locations that user *x* has been to over the past 14 days. The risk index uses a spatial transmission trajectory generated for each user from GPS data, rather than calculating an assumed probability of infection during each contact. It, therefore, provides a richer description of epidemic transmission than a traditional susceptible, infected, recovered model. This solves the problem of precise contact tracing.

The STRONG strategy (see Figure 1) will enable accurate location and tracking of sources of infection in the population and offer protection for the most susceptible individuals. In Figure 1, A shows a patient-focused biomedical data-based strategy, which centers on the source of infection and is unable to identify untraceable mild and asymptomatic patients in the population. B and C show the web-like individual tracking network built in STRONG for patients who are infected and the vast majority of the healthy population. D shows that STRONG can collect detailed, unique, permanent, and traceable spatiotemporal trajectory data for each user through social media and GPS–enabled smartphone technology, and use GeoAI algorithms to analyze data in real time. E shows the evolution of communication and transportation technology, resulting in the era of the Internet of Things, with high-speed dynamicity and high-dimensional complexity.

**Figure 1:**
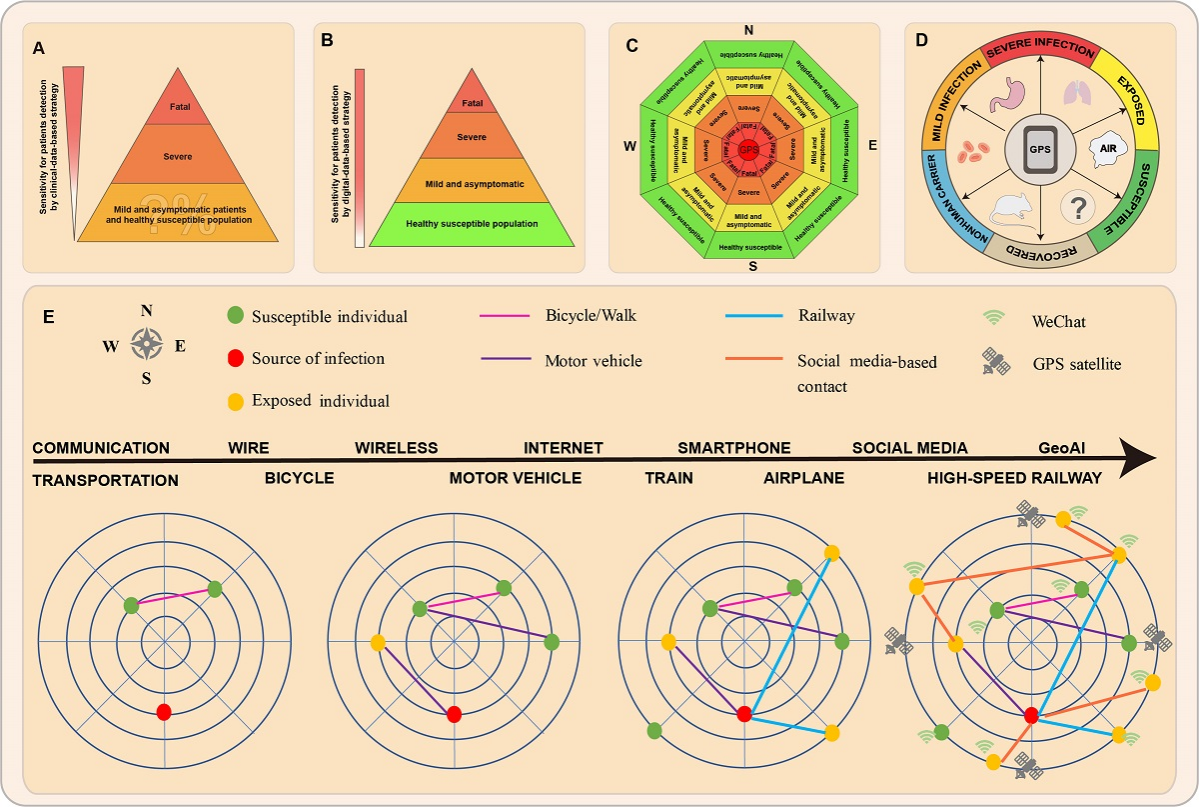
Comparison of patient-centered biomedical data-based strategy and digital data-oriented real time intelligent epidemic prevention and control strategy. GeoAI: geospatial artificial intelligence.

Our mini-program Geo-WAS was published on January 31, 2020. In March 2020, the Chinese government implemented a national monitoring system, using mobile phone base station positioning data to generate health QR codes as proof of an individual’s health condition. This system allowed people with the green QR code to return to work or school and even travel between different areas or provinces; this promoted the resumption of work, helped the precise control and smart management of COVID-19, and successfully prevented further COVID-19 transmission in China. Base station positioning data is not precise enough to distinguish all the risk contact compared with GPS data. However, for GPS data, a major unresolved issue is “noise” and false positive or negative identification; thus, the proper time and distance threshold to identify a risk contact or risk area in a GPS–based system still remains to be tested and adjusted in future practice. Similar applications of mobile health (mHealth) for COVID-19 such as “Tracetogether” from Singapore, “Aarogya Setu” from India, or “Big Sensor Data” use Bluetooth or SMS to help monitor contact in a different way [15-18]. Some technology firms like Apple and Google also cooperated to develop similar Bluetooth-based apps in iOS and Android systems. Compared with these apps still in development or just into use, which regard the smartphone as a simple radar, some mature ecology-formed platforms like WeChat and Alipay in China have already integrated multiple types of data including social communication, mobile payment, online community, and sound privacy agreements, and could, thus, gather traceable data both from the virtual and real world.

Though the mHealth–related products have been recently developing quickly, these apps rely on users or health care workers to report suspicious or confirmed COVID-19 cases on the system to update the risk area timely. There are concerns about personal confidentiality and privacy [19]. First, it has become acceptable to authorize the use of personal data including personal information, activity status data, and spatiotemporal data in the use of social media apps and other apps like Uber. However, the tension between proliferation of digital health care data and data privacy concerns is important because of the implications of any data breach or loss of health information. Some recently proposed novel protocols improving data transmission procedure could not satisfy complex data and further exploration is needed [16,20]. Blockchain technology may be helpful in managing this tension. This is a distributed database that originated from financial instrument research involving cryptocurrencies, of which bitcoin was the first well-established example, and could provide a reliable method for cross-platform management and sharing of medical data while ensuring its confidentiality [21]. Using blockchain technology, cryptographic health and operations data can be transferred from node to node by an automatically generated smart contract, a software protocol for legal agreements. It may also be worth considering people’s acceptance of the data privacy implications of Geo-WAS or the health QR code in light of the threat from COVID-19 to personal and public health. Second, collection, interpretation, and publication of information in a professional and appropriate way remain challenging. Smartphone social media apps are widely used, and it would not be an exaggeration to regard smartphones as essential for a substantial portion of the world’s population. Their use for health surveillance, therefore, goes beyond informing clinical staff and requires cooperation of an interdisciplinary team of biologists, clinicians, data scientists, and engineers. This could help to avoid misunderstandings of results or stigmatizing communities or individuals. Third, the dilemma between individual risk and population benefit is complex and varies in different areas and countries. It is an important part of public health ethics. To protect individuals and countries from the threat of COVID-19 infection and considering the necessity for policy changes that would interrupt personal lives and basic city functions, government should consider the use of STRONG, which will require recruitment of experts, close supervision of the process, and timely feedback.

In summary, STRONG is potentially a powerful strategy for epidemic prevention and control supported by smartphone social media app-based spatiotemporal trajectory data collection, big data compilation from multiple sources, and GeoAI–based real time data analysis. We have developed and published a suitable mini-program, Geo-WAS, and collected data for further analysis. There are, however, a number of ethical and technical challenges remaining, and we have not yet carried out a formal pilot study. However, during the current COVID-19 epidemic, similar health codes from other sources have been used to control the COVID-19 transmission without disrupting the regular social order [22].

## Abbreviations

AI: artificial intelligence
COVID-19: coronavirus disease
GeoAI: geospatial artificial intelligence
Geo-WAS: Geo WeChat AI System
GIS: geographic information systems
mHealth: mobile health
QR: Quick Response
R_0_: basic reproductive number
SARS-CoV-2: severe acute respiratory syndrome coronavirus 2
STRONG: spatiotemporal reporting over network and GPS
2019-nCoV: novel coronavirus
